# PaCS Is a Novel Cytoplasmic Structure Containing Functional Proteasome and Inducible by Cytokines/Trophic Factors

**DOI:** 10.1371/journal.pone.0082560

**Published:** 2013-12-17

**Authors:** Patrizia Sommi, Vittorio Necchi, Agostina Vitali, Daniela Montagna, Ada De Luigi, Mario Salmona, Vittorio Ricci, Enrico Solcia

**Affiliations:** 1 Department of Molecular Medicine, University of Pavia and Fondazione IRCCS Policlinico San Matteo, Pavia, Italy; 2 Pathologic Anatomy Service, University of Pavia and Fondazione IRCCS Policlinico San Matteo, Pavia, Italy; 3 Centro Grandi Strumenti, University of Pavia and Fondazione IRCCS Policlinico San Matteo, Pavia, Italy; 4 Pediatric Hematology/Oncology Service, University of Pavia and Fondazione IRCCS Policlinico San Matteo, Pavia, Italy; 5 IRCCS Istituto di Ricerche Farmacologiche “Mario Negri”, Milan, Italy; IISER-TVM, India

## Abstract

A variety of ubiquitinated protein-containing cytoplasmic structures has been reported, from aggresomes to aggresome-like induced structures/sequestosomes or particle-rich cytoplasmic structures (PaCSs) that we recently observed in some human diseases. Nevertheless, the morphological and cytochemical patterns of the different structures remain largely unknown thus jeopardizing their univocal identification. Here, we show that PaCSs resulted from proteasome and polyubiquitinated protein accumulation into well-demarcated, membrane-free, cytoskeleton-poor areas enriched in glycogen and glycosaminoglycans. A major requirement for PaCS detection by either electron or confocal microscopy was the addition of osmium to aldehyde fixatives. However, by analyzing living cells, we found that proteasome chymotrypsin-like activity concentrated in well-defined cytoplasmic structures identified as PaCSs by ultrastructural morphology and immunocytochemistry of the same cells. PaCSs differed ultrastructurally and cytochemically from sequestosomes which may coexist with PaCSs. In human dendritic or natural killer cells, PaCSs were induced in vitro by cytokines/trophic factors during differentiation/activation from blood progenitors. Our results provide evidence that PaCS is indeed a novel distinctive cytoplasmic structure which may play a critical role in the ubiquitin–proteasome system response to immune, infectious or proneoplastic stimuli.

## Introduction

Formation of cytosolic aggregates of ubiquitinated proteins is a hallmark of many severe human diseases involving the nervous system, skeletal muscle, heart and liver [1]–[3]. Classical aggresomes result from centripetal migration of small aggregates of misfolded proteins towards the microtubule-organizing center, in a microtubule- and dynein-dependent manner, to form juxtanuclear bodies enveloped by a cage of vimentin [4], [5]. In addition, a variety of discrete cytoplasmic structures accumulating ubiquitinated proteins have been described. Aggresome-like induced structures (ALISs) are cytosolic aggregates of ubiquitinated proteins induced in epithelial and non-epithelial cells *in vitro* under different stressful conditions that alter the quality control of endogenous or exogenous, natural or mutated, misfolded proteins [6]. The term ALIS derives from DALIS (dendritic cell aggresome-like induced structure) to indicate that such structures are not unique to dendritic cells (DCs). In DCs, DALISs form under bacterial lipopolysaccharide (LPS) stimulation as an accumulation of polyubiquitinated proteins prior to degradation, and may act as an antigen-storage compartment during cell maturation [7], [8].

Unlike aggresomes, ALISs are transient structures that are not localized in the pericentriolar area nor caged with vimentin, and usually do not accumulate proteasome [6], [9]. ALISs are indistinguishable cytochemically from sequestosomes [10], [11], and are mostly membrane-free, cytoplasmic inclusion bodies that contain ubiquitinated protein aggregates and p62 protein (also known as sequestosome 1). p62, together with autophagy-linked FYVE (ALFY) and NBR1 proteins, is required for ALIS formation and degradation by autophagy [11]–[13].

While investigating the formation of cytosolic misfolded protein inclusions in cultured yeast cells, Kaganovich et al. [14] found that soluble ubiquitinated proteins accumulated in a juxtanuclear compartment (named JUNQ, for juxtanuclear quality control). Proteasome was also concentrated in JUNQ, whereas insoluble proteins accumulated in a proteasome-negative peripheral perivacuolar compartment, the insoluble protein deposit (IPOD). Corresponding structures were also seen in cultured mammalian cells [14].

We recently described a cytoplasmic structure characterized by accumulation of cylindrical particles (∼13 nm thick and 14–40 nm long) and selective concentration of polyubiquitinated proteins and proteasome components [15]. This ubiquitin–proteasome-containing particle-rich cytoplasmic structure (PaCS) was first observed in human gastric epithelium infected with *Helicobacter pylori*. The PaCSs also showed selective concentration of *H. pylori* virulence factors VacA, CagA and urease, and intracellular NOD1 receptor for bacterial proteoglycans, in addition to basic dye metachromasia suggestive of the presence of anionic polysaccharides [15]. PaCSs were subsequently also detected in human gastric cancer cells, and although in the absence of *H. pylori* products, in a variety of other epithelial neoplasms [16]. In addition, PaCSs have been observed in neutrophils of patients with Shwachman–Diamond syndrome due to mutation of the *SBDS* gene involved in ribosome biogenesis and function [17], and in platelets and megakaryocytes of another genetic disease, *ANKRD26* gene-mutated thrombocytopenia [18].

The relationship between PaCSs, mainly observed in ex vivo pathological samples at transmission electron microscopy (TEM), and sequestosomes/ALISs or DALISs, and JUNQ or IPOD, all found mostly at confocal microscopy in a variety of cell lines and experimental conditions, remains unclear. In addition, the precise intracellular origin of PaCS and the nature of their inducing factors are unknown. Therefore, in the present study, we: (1) searched for PaCSs in cell lines reported to develop sequestosomes, ALISs or DALISs; (2) characterized the different cytoplasmic structures ultrastructurally and cytochemically using TEM, confocal microscopy and correlative TEM/confocal microscopy, with special reference to their content of ubiquitin–proteasome system (UPS) components, p62 protein and polysaccharides; and (3) investigated the possibility of inducing PaCSs in human immunocompetent cells *in vitro* under pertinent differentiation stimuli.

## Results

### PaCSs in epithelial, myeloid and neuroblastic cell lines

**Figure 1:**
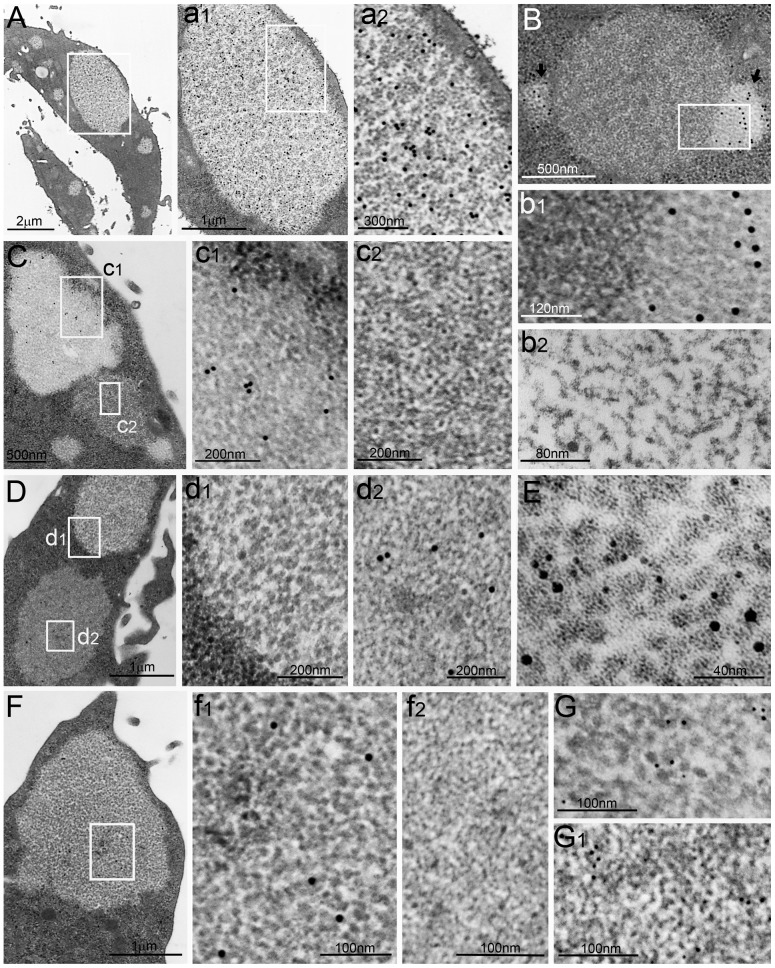
PaCSs and sequestosomes in HeLa cells. (**A**) Several PaCSs are scattered in the cytoplasm of two cells, the larger one (boxed) is enlarged in (**a1**) and further in (**a2**) to show particle accumulation in a clear cytosolic background and selective FK1 immunogold reactivity for polyubiquitinated proteins. (**B**) Two small PaCSs (arrows) adjacent to a large central sequestosome in a ribosome-rich cytosol; the boxed area is enlarged (**b1**) to show PaCS 20S proteasome reactivity (right) and non-reactivity of the sequestosome (left), characterized by amorphous to thinly granular material often forming short fibrils. The curved fibrils are better seen at higher magnification (**b2**) of a sequestosome with poorly contrasted amorphous interfibrillary material. (**C**) Three PaCSs surrounding a sequestosome; the larger PaCS enlarged in (**c1**) shows 19S proteasome immunoreactivity, which is missing in sequestosome (**c2**), whose thin granules are often aligned to form beaded fibrils. (**D**) PaCS (top) and sequestosome (bottom) in ribosome-rich cytoplasm, enlarged in (**d1**) and (**d2**), respectively, to show sequestosome p62 protein immunoreactivity and PaCS non-reactivity; ribosomes in the left lower corner of (**d1**). (**E**) High resolution micrograph of PaCS particles reactive for 20S proteasome (10 nm gold) and FK1 (5 nm gold) antibodies. Some particles were aligned end-on to form 40-nm-long cylinders. (**F**) Sparse glycogen immunoreactivity of a PaCS, enlarged (**f1**) to be compared with a glycogen-unreactive granular–fibrillary sequestosome (**f2**) of the same section. The anti-ubiquitin Z0498 antibody reacted with both PaCS (**G**) and sequestosome (**G1**).

**Figure 2:**
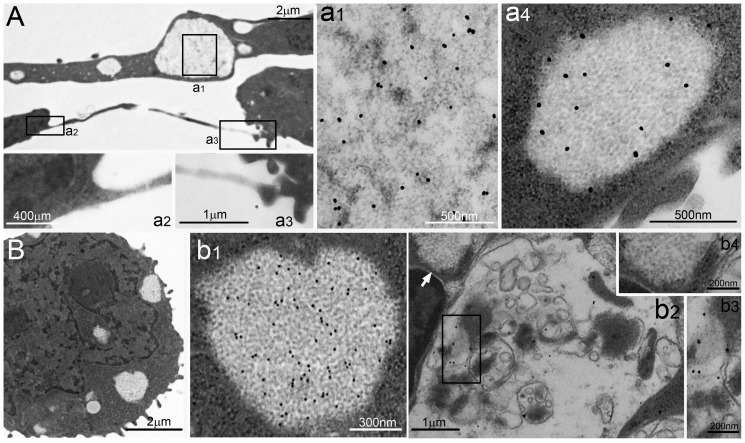
PaCSs in SH-SY5Y and HL-60 cells. (**A**) Ultrastructure of a neuroblastoma SH-SY5Y cell with characteristic PaCSs, enlarged (**a1**) to show relatively spaced particles and selective FK1 antibody immunogold. In (**A**) a neural cell process whose hillock-like origin and terminal button abutted on another cell is enlarged in (**a2**) and (**a3**), respectively. In (**a4**) a 20S proteasome-reactive PaCS from a different SH-SY5Y cell is filled with particles and surrounded by ribosomes. (**B**) Several PaCSs in an HL-60 cell; one of which (**b1**) shows FK1 immunogold; in (**b2**) ALFY reactivity of an autophagic vesicle, enlarged in (**b3**), and non-reactivity of a small PaCS (arrow), enlarged in (**b4**).

Cytoplasmic structures formed by cuboid to cylindrical particles, 12–14 nm thick and 14–20 (sometimes up to ∼40 nm long) thus reproducing the ultrastructure of previously described PaCSs, were detected by TEM in several neoplastic cell lines, including epithelial HeLa, AGS and Caco-2, HL-60 promyelocytic cells and SH-SY5Y neuroblastic cells when cultivated under basal conditions (Figures 1, 2). In most cases, PaCSs were found in a large proportion of cells, for example, 50–60% of HeLa cells. PaCSs were not found at all, or only in <1% of cells in MDA-MB-231 breast cancer cells, Jurkat T-cell lymphoma cells, murine RAW 264.7 neoplastic macrophages, monkey COS-7 neoplastic fibroblasts, primary human non-neoplastic fibroblasts, and nucleated blood cells. The PaCS particles were less osmiophilic than ribosomes and were embedded in a relatively clear space with little, if any, cytoskeletal network (Figure 1). Therefore, usually the PaCSs were distinguishable from the ribosome-rich cytoplasm as a clearer area ranging from 100 nm up to 5 µm in diameter. The electron density of the particles was generally uniform inside each PaCS, but changed from one cell to another and even from one PaCS to another inside the same cell. Sometimes, particle dissolution leaving a clear space with spotted remnants of amorphous material was seen. We observed patterns suggestive for end-on apposition of individual cylindrical particles (Figure 1E), as shown in 20S proteasome particles *in vitro* [19].

The immunogold procedures applied to aldehyde–osmium-fixed resin sections showed selective PaCS reactivity for FK1 antibody, specific for polyubiquitinated proteins [20], ubiquitin-directed Z0498 antibody, proteasome 20S and 19S antibodies, and specific glycogen antibody [21], [22], but not for p62 protein antibodies (Figure 1A–G). High-resolution immunogold labeling revealed selective reactivity of PaCS particles for 20S proteasome and polyubiquitinated proteins (Figure 1E).

### Sequestosomes and PaCSs are different structures

Another type of cytoplasmic structure (here provisionally called B-structure) was observed regularly in HeLa and MKN 28 cells cultivated under basal conditions, rarely in HL-60 cells, and not at all in SH-SY5Y, Jurkat, RAW 264.7, COS-7 cells, human fibroblasts or blood cells. In HeLa cells (Figure 1B–G), where the B-structure was found in 20–30% of cells, it was 0.3–4 µm in diameter and characterized by an amorphous to thinly granular and fibrillary substructure. This closely resembled that previously reported by Simonsen et al. [23] and Bjørkøy et al. [10] for p62-positive sequestosomes under basal or, more frequently, stressful conditions, as well as the filamentous structure we observed in *H. pylori*-infected human gastric epithelium [15]. The thin (5–7 nm thick) fibrils resulted from alignment of small granules (5–7 nm) to form curvilinear beaded fibrils that were visible with high-resolution TEM, depending from preservation of the amorphous component (Figure 1b1,b2),. In keeping with its ultrastructural similarity to the sequestosomes, the B-structure showed immunogold reactivity with p62 and ubiquitin Z0498 antibodies, whereas unlike PaCSs, it failed to react with FK1, 20S or 19S proteasome and glycogen antibodies (Figure 1B–G). Therefore, from now on we will refer to such a structure as granular–fibrillary sequestosome.

PaCSs and granular–fibrillary sequestosomes were frequently adjacent to each other or even in direct continuity, although with limited mixing of their respective contents (Figure 1B). Both were preferentially distributed in ribosome-rich cytoplasm, with or without associated RER cisternae, which, remained outside the structure core, as did other organelles, including mitochondria, endosomes, lysosomes, Golgi, and most background cytoskeletal network. Neither PaCSs nor sequestosomes had direct contact with the plasma membrane, from which they were always separated at least by a band of cytoskeleton-rich cytoplasm. Usually, no peripheral limiting membrane was seen around PaCSs or most of the sequestosomes. However, a few of the latter showed such membranes, often coupled with irregular osmiophilic contents suggestive of autophagic vesicles and with reactivity for ALFY protein, which commonly labeled autophagic structures while being unreactive with PaCSs (Figure 2b2–b4).

### Aldehyde–osmium fixation is required for PaCS detection by TEM or confocal microscopy

**Figure 3:**
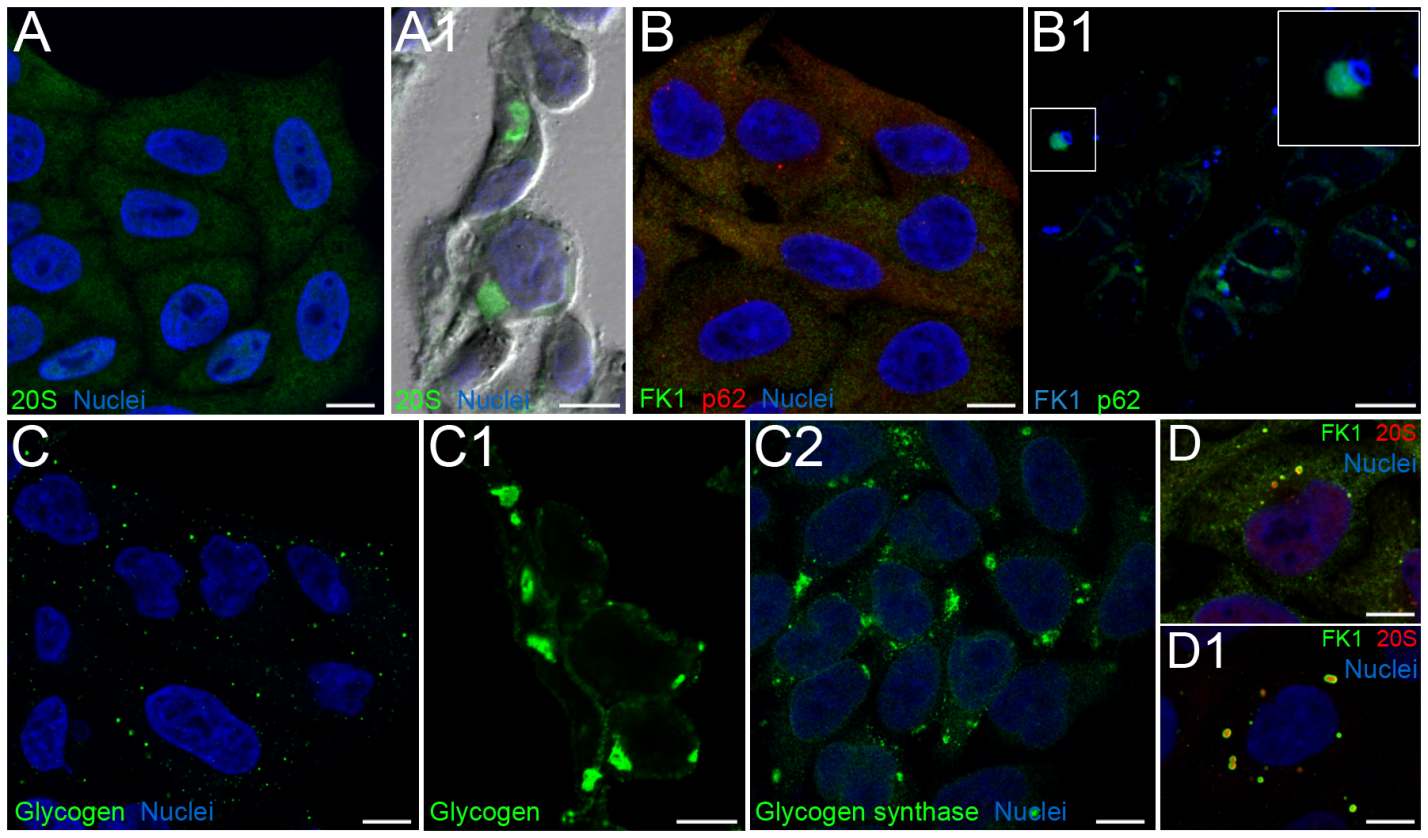
Detection of PaCSs by confocal microscopy. (**A**) Only weak diffuse 20S proteasome immunofluorescence was seen in control HeLa cells after standard preparation (i.e., 15 min paraformaldehyde fixation), compared to the large 20S proteasome-reactive structures visible after aldehyde–osmium fixation and paraffin-embedding (**A1**, immunofluorescence and phase-contrast overlay). (**B**) Poor FK1 reactivity (green) and a few p62-reactive (red) spots were seen in the cytoplasm of standard-prepared HeLa cells, whereas after aldehyde–osmium fixation, large FK1-positive (blue) structures appeared (**B1**), lacking colocalization with p62 green fluorescence despite occasional juxtaposition (inset). (**C**) Only scattered minute glycogen deposits appeared in standard-prepared cells, whereas large deposits were seen after aldehyde–osmium fixation (**C1**); large deposits of glycogen synthase were seen after methanol fixation (**C2**). Standard confocal microscopy of epoxomicin-treated (**D**) or puromycin-treated (**D1**) HeLa cells showed prominent FK1/20S proteasome-reactive cytoplasmic bodies. Bars, 10 µm.

When antibodies reacting selectively with PaCSs in TEM preparations were applied to standard confocal microscopy specimens of HeLa or other cells, only weak diffuse staining or scattered minute fluorescent spots were seen (Figure 3A,B). However, when aldehyde–osmium-fixed paraffin sections or semithin (1 µm thick) sections from aldehyde–osmium-fixed resin blocks for TEM were immunostained, we observed numerous fluorescent cytoplasmic bodies (0.2–5 µm diameter) (Figures 3A1, 3B1, 4A, 4B) comparable with PaCSs seen by TEM. The glycogen antibody behaved like FK1 or proteasome antibodies with respect to fixatives (Figure 3C,C1), while glycogen synthase, which colocalizes with glycogen inside the “glycosome” [24], also showed PaCS immunoreactivity in methanol/acetone fixed/permeabilized cells (Figure 3C2). Parallel ultrastructural investigation of PaCSs from aldehyde-fixed specimens in the absence of osmium revealed marked loss of their constitutive particles, with scarce preservation of UPS immunoreactivity (data not shown). Thus, it was concluded that for cells cultivated in basal conditions combined aldehyde-osmium fixation is essential for effective morphologic and cytochemical preservation of PaCSs. In contrast, as already shown for sequestosomes/ALISs [6], [10], standard confocal microscopy preparations were effective for detecting cytoplasmic structures reactive for PaCS markers in cells treated with the proteasome inhibitor epoxomicin or the premature protein chain terminator puromycin (Figure 3D,D1), thus suggesting that the treatments substantially reduced PaCS proteins solubility favoring their aggregation and precipitation.

**Figure 4:**
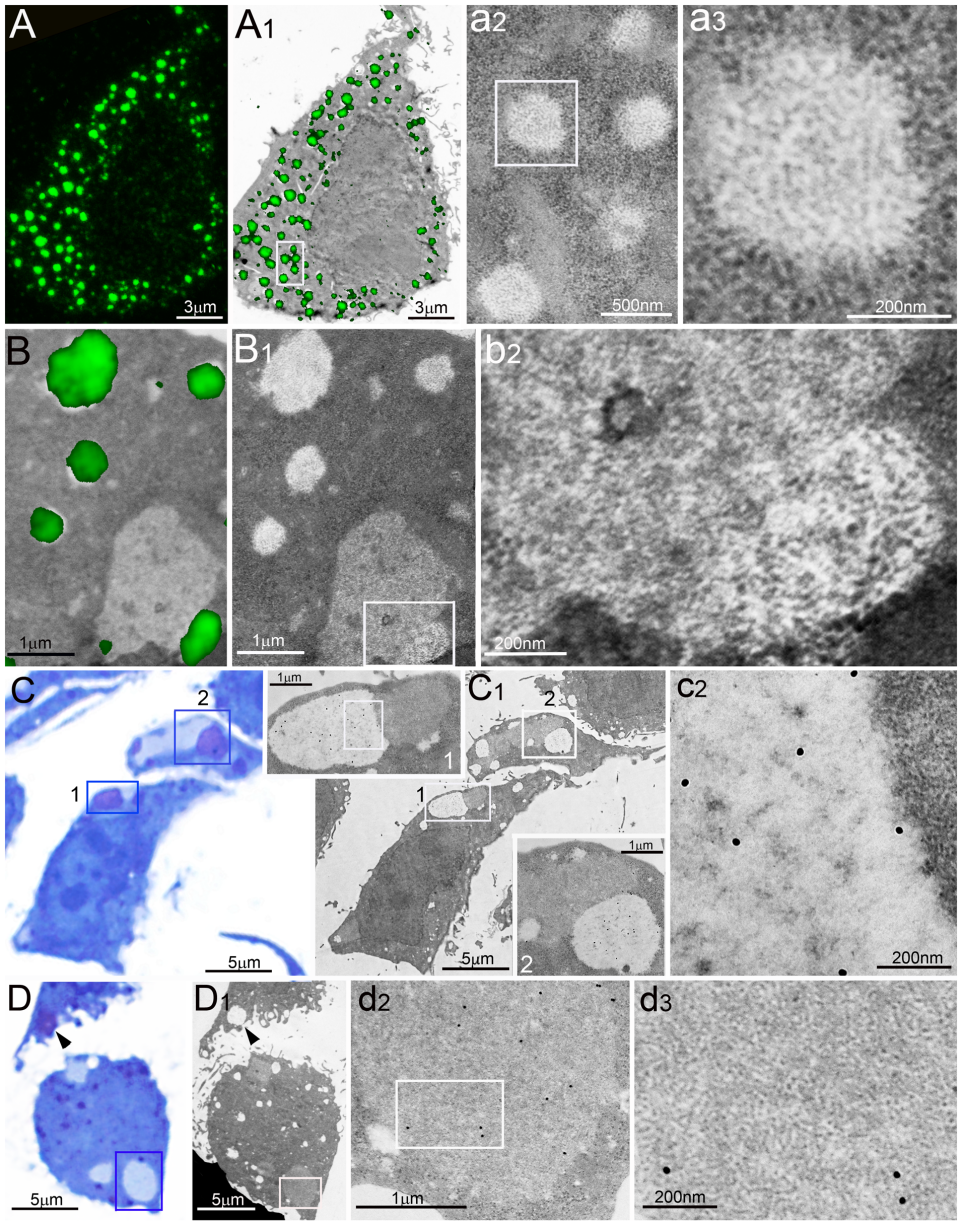
Correlative light/electron microscopy of PaCSs. (**A**, **B**) Direct correlation between confocal microscopy immunofluorescence and TEM in an aldehyde–osmium fixed HeLa cell. (**A**) 20S proteasome immunofluorescence (green) of numerous cytoplasmic bodies, projected on the corresponding TEM micrograph (**A1**) to show overlapping of proteasome immunofluorescence spots with cytoplasmic PaCSs; a few of which are enlarged in (**a2**) and further in (**a3**) to show their distinctive ultrastructure. (**B**) Combined immunofluorescence/TEM image showing several proteasome-reactive PaCSs (green) and a large proteasome-unreactive sequestosome, as shown by TEM alone in (**B1**); part of the sequestosome and an adhering PaCS are enlarged (**b2**) to show their distinctive ultrastructure. (**C** and **D**) Direct identification of metachromatic bodies with PaCSs using consecutive semithin (light microscopy)/thin (TEM) section analysis from aldehyde–osmium-fixed, resin-embedded HeLa cells. Toluidine blue staining of a semithin section shows red–violet metachromatic bodies (**C**), corresponding to clear areas in a consecutive TEM section (**C1**). Boxed areas are enlarged in insets 1 (further enlarged in **c2**) and 2 to magnify 20S proteasome immunogold, besides faintly contrasted PaCS-type particles. Note the weak grey–blue staining (**C**) and the heavier electron density (**C1**) of the two sequestosomes adhering to boxed PaCSs and showing granular–fibrillary ultrastructure (**c2**) (right upper corner). (**D** and **D1**) Several small PaCSs in the bottom cell and a larger one in the upper cell (arrowhead) heavily stained by toluidine blue (**D**), while three sequestosomes were lightly stained. Most of such bodies were also identified by TEM in a consecutive thin section (**D1**), where PaCSs appeared as clear spots and sequestosomes as areas with intermediate electron density. The largest sequestosome is enlarged in (**d2**) and (**d3**) to magnify its thin granular–fibrillary ultrastructure and p62 protein immunoreactivity.

To ascertain that confocal microscopy immunofluorescent bodies of aldehyde–osmium-fixed sections were bona fide PaCSs, correlative light and electron microscopy studies were carried out. In particular, correlative TEM immunogold/confocal immunofluorescence analyses performed on opposite sides of the same HeLa cell resin thin section (70 nm thick) allowed us to characterize UPS-reactive PaCSs and p62-reactive sequestosomes as separate cytoplasmic structures (Figure 4A,B).

**Figure 5:**
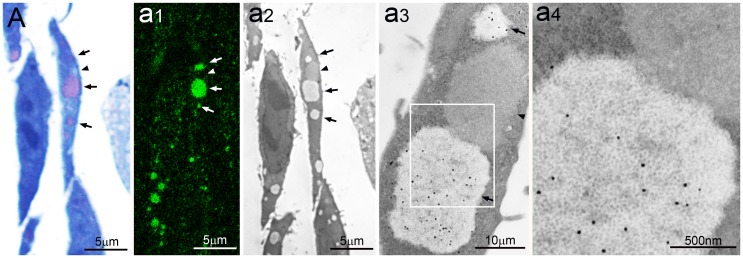
PaCSs are metachromatic and chondroitin sulfate-positive bodies. (**A**) Toluidine blue metachromatic bodies (arrows) corresponding to chondroitin sulfate immunofluorescent bodies (**a1**), and TEM-characterized PaCSs (**a2–4**) in consecutive aldehyde–osmium-fixed resin sections of HeLa cells. A sequestosome (arrowhead) lightly stained (**A**), unreactive for chondroitin sulfate (**a1**) and moderately electron dense (**a2**) is enlarged in (**a3**) and (**a4**) to show its distinctive ultrastructure and unreactivity for FK1 immunogold, which selectively labeled the adjacent particle-filled PaCS.

Semithin aldehyde–osmium-fixed resin sections stained with toluidine blue showed selective red–violet metachromatic staining of cytoplasmic areas (Figure 4C,D). When these were viewed in consecutive thin sections by TEM, they were identified as PaCSs due to their distinctive particles, as well as proteasome and FK1 immunogold reactivity coupled with p62 non-reactivity (Figure 4C1,c2,D1,d2,d3). In the same or consecutive sections, granular–fibrillary sequestosomes were also identified by TEM. They were found to react with p62 antibodies and to lack basic dye metachromasia, while staining weak grey–blue with toluidine blue under light microscopy (Table 1). PaCS metachromasia is abolished at acidic pH, suggesting the involvement of anionic glycoconjugates such as proteoglycans [15], [16], therefore, we also tested glycosaminoglycan-directed antibodies. As shown in Figure 5, the chondroitin sulfate antibody CS-56 reacted with metachromatic bodies that corresponded to PaCSs in consecutive thin sections viewed under TEM, while adjacent sequestosomes remained negative for chondroitin sulfate.

**Table 1:** Differential patterns of PaCSs and sequestosomes in HeLa cells^a^.

	*PaCS*	*Sequestosome*
***Light microscopy:***		
*Toluidine blue*	Red–violet	Weak grey–blue
***Ultrastructure:***	Barrel-like particles	Amorphous to granular with curvilinear beaded fibrils
***Confocal microscopy and ultrastructural immunocytochemistry:***		
*Polyubiquitinated proteins (FK1)*	+	−
*Ubiquitin*	+	+
*Proteasome*	+	−
*Glycogen*	+	−
*Chondroitin sulfate*	+	−
*p62*	−	+

–osmium-fixed cells.^a^ Aldehyde

### PaCSs contain functional proteasome

**Figure 6:**
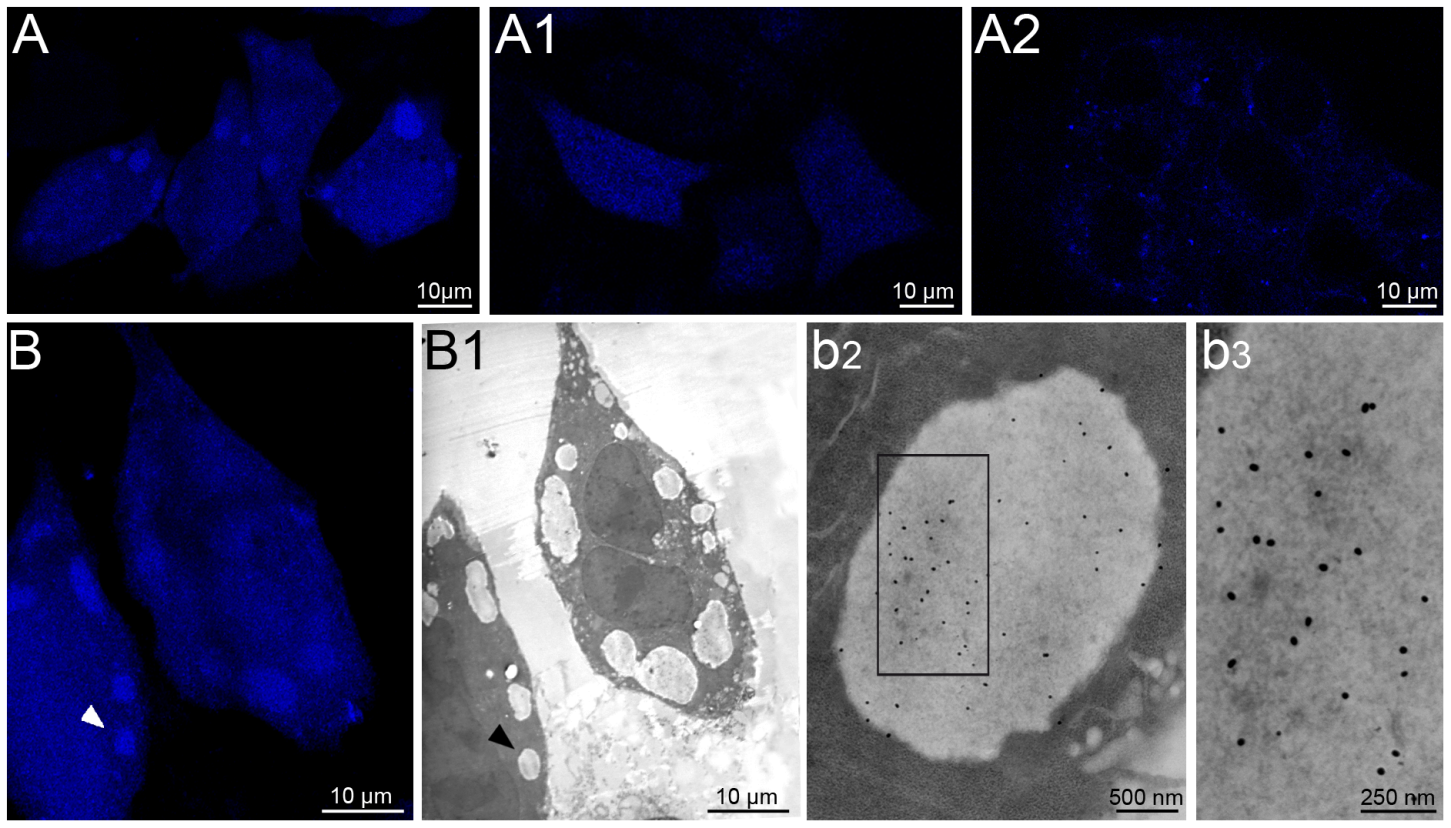
Proteasome activity in PaCSs of living cells. (**A**) Proteasome chimotrypsin-like activity shown by TED peptide cleavage in living HeLa cells concentrated in cytoplasmic bodies resembling PaCSs in size and intracellular distribution, and (**A1**) was greatly reduced by epoxomicin treatment. (**A2**) No comparable fluorescent areas appeared in the cytoplasm of TED-incubated living COS-7 cells. (**B**) TED-induced proteasome fluorescent bodies in two living HeLa cells under confocal microscopy corresponding in an aldehyde–osmium-fixed resin TEM section of the same cells (**B1**) to clear spots identified as PaCSs at higher resolution, owing to their faintly contrasted barrel-like particles and selective FK1 immunoreactivity, as shown in (**b2**) and (**b3**) for the one arrowhead in (**B**) and (**B1**).

To investigate whether PaCSs accumulate functionally active proteasome and to rule out the possibility that PaCSs are procedural artifacts, we analyzed living cells under confocal microscopy and correlative confocal/electron microscopy. We exploited a recently developed tool to evaluate proteasome chymotrypsin-like activity in living cells, based on selective cleavage of an internally-quenched TAT-EDANS-DABCYL (TED) peptide, which readily penetrates cell membranes and becomes fluorescent when selectively degraded by the proteasome machinery [25]. Confocal microscopy of living HeLa cells incubated with TED peptide showed fluorescent cytoplasmic areas, progressively increasing in intensity and closely resembling proteasome-immunofluorescent PaCSs as found in aldehyde–osmium fixed cells (Figure 6A). Treatment with epoxomicin, a selective inhibitor of proteasome proteolytic activity, greatly reduced TED-dependent fluorescence (Figure 6A1), further confirming the role of the proteasome in the development of PaCS-like fluorescence. Correlative confocal/electron microscopy of the same cells enabled us to demonstrate directly that TED-induced cytoplasmic fluorescence corresponded to FK1-positive PaCSs (Figure 6B-b3). There was no fluorescence in the cytoplasm of TED-treated living COS-7 cells (Figure 6A2), which lacked PaCSs by parallel TEM investigation. Thus, correlative confocal/TEM analysis of TED-treated HeLa cells enabled us to confirm directly the PaCS nature of TED-dependent fluorescent cytoplasm by both ultrastructural morphology and immunocytochemistry, and to demonstrate the presence of PaCSs in living cells.

### PaCSs induction in human DCs and natural killer cells

Cytosolic aggregates of polyubiquitinated proteins, named DALISs, have been described in murine DCs by confocal microscopy [8] and proteasome has been detected by immunofluorescence and immunogold TEM in poorly defined mucoid masses of murine natural killer (NK) cells [26]. Both DCs and NK cells are known to originate *in vitro* from mononuclear cells after treatment with appropriate cytokines/trophic factors [27], [28]. Therefore, we decided to investigate human DCs and NK cells as well as their blood precursors for PaCSs.

**Figure 7:**
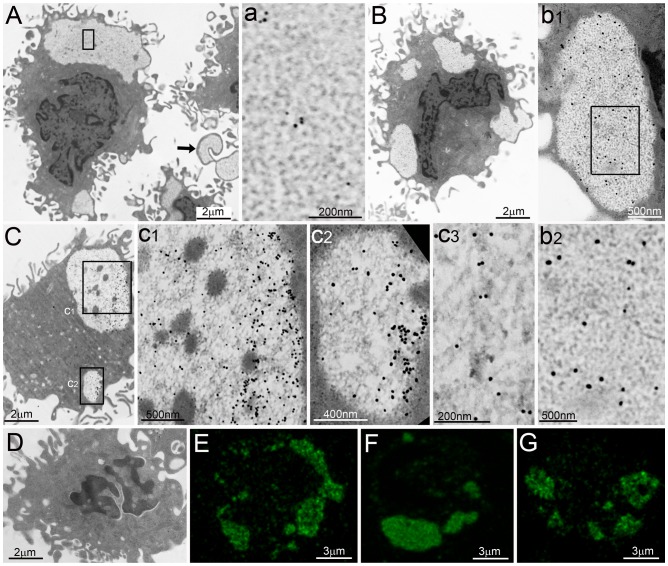
PaCS in human DCs. (**A**) Several PaCS-filled blebs (arrow) and intracytoplasmic PaCSs; the largest of which is enlarged (**a**) to show barrel-like particles and sparse 19S proteasome immunoreactivity. (**B**) Four PaCSs, one enlarged in (**b1**) and further in (**b2**) to show particles FK1 reactivity. (**C**) Two PaCSs, enlarged in (**c1**) and (**c2**), showed glycogen immunoreactivity polarized on the right side; note that PaCS particles were not polarized and that glycogen immunogold deposits were frequently unrelated to them. Selective 20S proteasome reactivity of PaCS particles from another section of the same cell is shown in (**c3**). (**c1**) Note residual round islets of cytoskeleton-rich cytoplasm inside cytoskeleton-poor PaCS – an uncommon finding. (**D**) An untreated blood monocyte lacking cytoplasmic PaCSs. Confocal microscopy of three aldehyde–osmium-fixed DCs showed immunofluorescence for proteasome (**E**), FK1 (**F**), and chondroitin sulfate (**G**).

PaCSs with characteristic barrel-like particles and reactivity for ubiquitin, polyubiquitinated proteins, proteasome and glycogen, but not p62 antibodies, were found by TEM in most DCs obtained from monocytes treated with granulocyte–macrophage colony-stimulating factor (GM-CSF) and interleukin (IL)-4 (Figure 7A–C). No PaCSs were observed in untreated monocytes (Figure 7D). Unlike UPS particles, glycogen immunogold reactivity sometimes showed obvious polarization inside PaCSs (Figure 7C,c1,c2), mimicking the glycogen intracellular polarization shown under light microscopy by tissues fixed in aqueous solutions and suggesting physical separation of at least part of glycogen molecules from UPS particles. No p62-positive sequestosome-type bodies with amorphous to granular–fibrillary content were observed. In addition, in aldehyde–osmium-fixed cells, confocal microscopy showed selective proteasome, polyubiquitinated proteins, and chondroitin sulfate immunofluorescent bodies (Figure 7E,G), comparable with those showing toluidine blue metachromasia.

**Figure 8:**
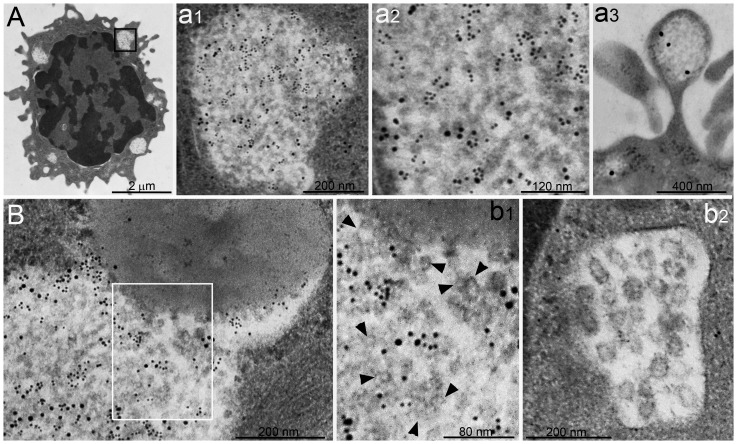
PaCSs in human NK cells. (**A**) IL-15-treated human NK cell showing several small PaCSs; one of which is enlarged in (**a1**) and further in (**a2**) to show barrel-like particles with FK1 (5 nm gold) and 20S proteasome (10 nm gold) immunoreactivity; (**a3**) a PaCS-filled bleb. (**B**) Part of a mixed lytic granule, enlarged in (**b1**), showing in its vesicular component both barrel-like particles (some of which had FK1 and/or 20S proteasome immunoreactivity) and unreactive vesicles (arrowheads). Note in the upper part of (**B**) and (**b1**) the unreactive dense core of the lytic granule. For comparison, a multivesicular body, unreactive for both FK1 and 20S proteasome antibodies, is shown in (**b2**), from another NK cell in the same section as in (**A**) and (**B**).

We observed by TEM human NK cells obtained in vitro from blood mononuclear cells, with or without subsequent overnight activation with IL-2 or IL-15. PaCSs with the usual barrel-like particles and reactivity for 20S proteasome, FK1 and glycogen antibodies were found in >20% of IL-treated cells; mostly in ribosome-rich cytoplasm and sometimes inside peripheral blebs (Figure 8A). No PaCSs were observed in untreated cells (not shown). On ultrastructural and immunocytochemical bases, it seems that at least some previously described proteasome-reactive mucoid masses correspond to PaCSs. PaCS-type particles immunoreactive for both 20S proteasome and FK1 antibodies were also detected in the vesicular component of composite lytic granules (Figure 8B, b1), in keeping with previous detection of proteasome [26]. In contrast, vesicles coexisting with PaCS-type particles inside such granules remained unreactive for UPS, as did the solid component of the granules and some multivesicular bodies found in the cytoplasm of NK cells (Figure 8B,b1,b2). No sequestosomes were found.

Thus, both human DCs and NK cells develop PaCSs during their differentiation/activation process under cytokine/trophic factor treatment.

## Discussion

This study shows that at least two types of ultrastructurally and cytochemically different ubiquitin-reactive cytoplasmic structures are present in several cultured cell lines under basal conditions: (1) PaCS, characterized by accumulation of cuboid to cylindrical particles and selective immunoreactivity for polyubiquitinated proteins and proteasome components, although unreactive for p62 protein; and (2) a p62-reactive structure, characterized by deposition of amorphous to thinly granular–fibrillary material, reactive for ubiquitin and unreactive for proteasome. The latter structure was previously described by confocal and electron microscopy [10], [23] as p62 bodies or sequestosomes *in vitro*, and by Denk et al. [29] as p62-positive hepatocellular hyaline bodies *in vivo*. It also corresponds to the ALIS observed by confocal microscopy only in various cell lines under stressful conditions [6]. In contrast, PaCSs were characterized only very recently; mostly in human pathological tissues [15], [16]. Kaganovich et al. [14] described the proteasome-negative IPODs and the JUNQs, juxtanuclear bodies characterized by immunoreactivity for both soluble ubiquitinated proteins and proteasome. In principle, their identification with sequestosomes and PaCS, respectively, seems likely; however the close topographic connection that we observed between sequestosomes and PaCSs in mammalian cells contrasts with the separation reported for IPODs and JUNQs. In addition, there may be a relationship between PaCSs and DALISs, the polyubiquitinated protein-reactive structure originally described by Lelouard et al. [7], [8] by confocal microscopy in murine DCs, although they did not observe proteasome in it and did not investigate its ultrastructure.

The curvilinear, ribbon-like, beaded fibrils in HeLa sequestosomes closely resembled in structure, thickness (5–7 nm) and length (100–200 nm) the oligomeric protofibrils described during *in vitro* fibrillization of Alzheimer amyloidogenic A protein by atomic force microscopy [30] or electron microscopy [31]. They also resemble Parkinson α-synuclein protein ([32] and the fibrils formed by huntingtin polyglutamine repeat sequences *in vitro* and *in vivo* [33]. Such protofibrils are formed by lining up of spherical particles [34], and both particles and protofibrils, but not fully formed amyloid fibrils, react with the conformational antibody A11, irrespective of their peptide sequence [35], [36]. The ultrastructural similarity of HeLa cells sequestosomes and intracellular deposits formed by amyloidogenic proteins calls attention on the known presence of viral oncoprotein oligomers, reactive with conformational amyloid stains (Thioflavin or Congo Red) in HPV-transformed cancer cells [37], [38], to which HeLa cells also belong [39]. It is also worth noting that structural analysis and molecular modeling have shown that p62 forms oligomeric chains with a “beads-on-a-string” structure [40], thus raising the possibility that p62 by itself may contribute to sequestosome ultrastructure.

It is generally agreed that sequestosomes, with their autophagy-promoting proteins p62, NBR1 and ALFY, are largely destined to autophagy [6], [10], [11], [13]. It has been proposed that protein oligomerization plays an important role in substrate selection for autophagy [41], and p62 protein self-oligomerization is essential for targeting it to the autophagosome formation site [42]. Thus, it is of particular interest that selective genetic suppression of autophagy in mice resulted in neurodegeneration with cytoplasmic accumulation of amorphous to fibrillary protein deposits [43], closely resembling sequestosome ultrastructure but clearly differing from PaCSs. Direct involvement of autophagy impairment in the genesis of sequestosomes/ALISs through p62-promoted protein aggregation is also suggested by recent findings in HeLa cells [44].

The apparent lack of PaCS identification and separation from sequestosomes in most previous studies may seem surprising. However, we found that a major requirement for PaCS detection was the addition of osmium to aldehyde fixatives, which is standard for TEM preparation but unusual for confocal microscopy procedures. Osmium tetroxide is a wide-spectrum fixative that preserves cellular components that escape aldehyde fixation, including polysaccharides such as glycogen and glycosaminoglycans and related conjugates [45]. Thus, the PaCS-preserving effect of osmium is not surprising, and it proved essential for our investigation despite its tendency to impair the reactivity of some epitopes in immunocytochemical tests. The effectiveness of our procedure was supported by correlative confocal/TEM microscopy of the same or consecutive aldehyde–osmium-fixed resin sections, which, combined with antibody colocalization tests, provided direct characterization of PaCSs and their assessment as novel cellular structures. Of particular relevance was the detection in living HeLa cells of proteasome chymotrypsin-like activity concentrated in cytoplasmic structures showing all morphological patterns of PaCSs (including intracellular distribution and cell type dependence), and indeed identified as PaCSs by TEM analysis of the same cells. This finding proves that PaCS is a structural component of living cells where proteasome function is concentrated, in addition to individual protein constituents.

PaCS origin and function remain to be fully elucidated. The smallest PaCSs invariably appear in close connection with ribosomes, from which they are likely to originate [15]. PaCS formation is associated with increased cellular content of both proteasome and polyubiquitinated proteins, as shown by whole cell lysate immunoblotting as well as compartment-selective TEM immunogold quantitation [17], [18]. In several cell lines, PaCS development follows cell differentiation/activation by trophic factors and cytokines, such as GM-CSF and IL-4 for DCs, IL-2 and IL-15 for NK cells, or thrombopoietin, IL-6 and IL-11 for megakaryocytes in type 2 thrombocytopenia [18]. This is in keeping with the EGF receptor overexpression and activation seen in two PaCS-developing conditions, pancreatic cystic adenoma [16], [46] and *H. pylori*-infected gastric epithelium [15], [47]. Thus, cell differentiation and activation seem to be involved, at least in several cases.

There is also evidence for enhanced proteasome biosynthesis secondary to proteasome functional impairment; a sort of feedback mechanism for restoration of adequate proteasome function [48]. An exaggerated response elicited by proteasome dysfunction would be in keeping with the accumulation of polyubiquitinated proteins in all PaCSs investigated, despite proteasome colocalization [15], [16 and this study] and overexpression [18], as well as with similar findings in neoplastic cells [49]. Concerning neoplastic cells, it has been suggested that the increased accumulation of ubiquitinated proteins causing UPS stress, rather than increased proteasome per se, renders such cells more sensitive to proteasome inhibitors, with resulting decreased proliferation and increased apoptosis [49].

As to the nature of the putative proteasome dysfunction, it is well known that it is associated with protein aggregation, which may precede actual protein deposition and is more likely to be caused by soluble protein oligomers [50]. Impaired delivery of ubiquitinated proteins to the proteasome, rather than impaired ubiquitination or proteasome proteolytic function per se, seems to be involved in some cases with a relative lack of 19S proteasome components [51]. In this context, it is of interest that the short (∼13 nm thick and 14–20 nm long), uniformly punctate, cylindrical, proteasome-reactive particles that are predominant inside most PaCSs resemble ultrastructurally the 20S core more than the full 26S proteasome particles, with their irregularly shaped caps [52], [53]. Longer (40–45 nm) and more irregular cylindrical particles, more like the complete 26S proteasome complex, are also seen inside PaCSs [15]. However, these forms are less common and, as far as length is concerned, could be partly accounted for by end-on concatemers of 20S particles [19], [54]. Hence, a relative lack of the fully active 26S molecular species required for degradation of polyubiquitinated proteins [55], [56] may be expected inside PaCSs. This might account for the accumulation of polyubiquitinated proteins as due to inefficiency of the 20S proteasome in their degradation, despite its capacity to ubiquitin-independently degrade short peptides, unstructured proteins [57], [58], or oxidized proteins [59]. More investigation of this issue is needed.

It has been reported that LPS, known to efficiently induce ALIS in macrophages [6], [60], interacts directly with proteasome which may contribute to LPS-induced inflammatory responses of macrophages [61], and that UPS components localized in lipid rafts may be instrumental to this action [62]. It should be noted that we never observed any direct physical contact between UPS-storing PaCSs and the plasma membrane, where lipid rafts are to be found, and that in macrophages we failed to detect PaCSs, even after LPS treatment. This renders unlikely a role of PaCSs in UPS-mediated macrophagic responses elicited by LPS. Nevertheless, it may be recalled that UPS components are normally scattered throughout the cytoplasm of unstimulated cells [63] and even, though at reduced concentration, in the cytoplasm of PaCS-bearing either *H. pylori*-infected, or mutated or cytokine-stimulated cells [15], [17], [18].

The presence of PaCS-filled blebs in DCs and NK cells (this study) as well as in megakaryocytes from *ANKRD26*-mutated type 2 thrombocytopenia [18] is of particular interest. Cytoplasmic bleb formation is an early sign of apoptosis [64], [65], and proteasome-filled cytoplasmic blebs (in association with actin-filament rearrangement) are seen in cells specifically induced to apoptosis through p53 activation [66]. However, no PaCS induction is seen in neutrophils undergoing apoptosis because of oxidative stress [17], and it seems unlikely that PaCS function is restricted to apoptosis; especially considering that most infected or neoplastic PaCS-storing cells lack actual signs of apoptosis [15]–[17]. A more general role of PaCS-filled blebs in intercellular communication may be considered. Indeed, it has been shown that cytoplasmic blebs released from apoptotic cells are taken up by DCs more efficiently than apoptotic cell bodies, and unlike the latter, induce cell maturation with increased co-stimulatory molecules and cytokine production, as well as allogenic T cell activation in coculture [65].

We showed that glycogen and chondroitin-sulfate-reactive anionic glycoconjugates are common components of PaCSs, which may explain their poor preservation in aqueous aldehyde fixatives, given the well-known high solubility of both polysaccharides. It should be recalled that large deposits of glycogen particles have long been reported in some cells by TEM, with special reference to clear-cell neoplasms [67], although UPS components were never tested in such structures. The presence of glycogen and glycogen synthase in PaCSs may be explained by several recently discovered relations between the UPS and glycogen-related proteins: (1) the UPS regulation of proteins such as laforin, acting in complex with E3 ligase malin [68], the protein targeting glycogen [69] and AMP kinase [70]; (2) direct involvement of the laforin–malin complex in misfolded protein degradation [71]; and (3) the recruitment of this complex in aggresome-like cytoplasmic structures, together with ubiquitin and glycogen [72]. In addition, a role has been proposed for Rab25/AKT-activated glycogen stores in promoting cancer cell survival through aerobic-glycolysis-mediated ATP synthesis, a crucial requirement for enhanced UPS function [73], [74].

The detection of chondroitin sulfate chains in PaCSs likely accounts for the toluidine blue metachromasia that we previously reported in PaCSs [15], [16]. It is also in keeping with the colocalization of heparan sulfate and related degradation products with 20S proteasome in oxidized misfolded protein deposits of N2a neuroblastoma cells [75], as well as with the detection of proteasome in mucoid masses of NK cells [26, this study]. The early observation that heparin and sulfated glycolipids specifically bind and functionally activate proteasome in vitro [76] should encourage interest in this topic, also considering that chondroitin sulfate chains can interact with and act as co-receptors for various growth factors [77], including EGF and FGF, which may be involved in the genesis of PaCSs.

In conclusion, PaCSs are novel distinctive structures where polyubiquitinated proteins and proteasome accumulate in a peculiar, highly soluble background enriched in polysaccharides. Several factors are likely to have a role in PaCS origin: (1) cell differentiation and activation by cytokines and trophic factors, as in cultured DCs, NK cells and *ANKRD26*-mutated megakariocytes; (2) cell activation and transformation by oncogenic microbial products, as in *H. pylori* gastritis [78] and HPV oncogene-expressing HeLa cells [39]; (3) leukemia-prone mutations, as in Shwachman–Diamond neutropenia [79] and *ANKRD26*-related thrombocytopenia [80], [81]; or (4) apparently non-mutated, constitutive overexpression of EGF receptor, as for pancreatic serous cystic adenoma [46]. PaCSs clearly differ from sequestosomes cytochemically and ultrastructurally, as well as for cell type distribution. However, in some cells they coexist and are closely related topographically, which suggests functional interaction. It may be speculated that the ubiquitin–proteasome-rich PaCSs, arising at the site of misfolded/mutated protein synthesis, mount a first degradation attempt. When this fails and high concentrations of aggregating proteins accumulate, a precipitation/sequestering process supervenes to remove them from the cytosol [82], predisposing the resulting sequestosomes to autophagy and lysosomal degradation [6], [10], [11], [14]. In some non-pathological cells that develop PaCSs in the absence of sequestosomes (e.g., DCs), PaCSs might fulfill more specific functions, including antigen storage, processing and release, as already proposed for DALIS [7], [8], [83]–[85].

## Materials and Methods

### Cells

We used the following cell lines: HeLa (ATCC CCL-2; from human cervix adenocarcinoma), AGS (ATCC CRL-1739; from human gastric adenocarcinoma), Caco-2 (ATCC HTB-37; from human colorectal adenocarcinoma), COS-7 (ATCC CRL-1651; monkey kidney SV40-transformed fibroblast-like cells), HL-60 (ATCC CCL-240; from human acute promyelocytic leukemia), Jurkat E6-1 (ATCC TIB-152; from human acute T-cell leukemia), MDA-MB-231 (ATCC HTB-26; from human metastatic breast adenocarcinoma), MKN 28 (from human well-differentiated gastric tubular adenocarcinoma [86]), RAW 264.7 (ATCC TIB-71; mouse macrophage from a tumor induced by Abelson murine leukemia virus), and SH-SY5Y (ATCC CRL-2266; from human metastatic neuroblastoma). Cells were grown in DMEM (except for Jurkat and HL-60 cells which were grown in RPMI 1640) supplemented with 10% FBS and 2 mM glutamine (all from Lonza, Basel, Switzerland) at 37°C in a humidified atmosphere of 5% CO_2_ in air. When specified, HeLa cells were incubated for 7 h with 5 µg/ml puromycin (Sigma–Aldrich, St. Louis, MO).

Immature human DCs were generated according to Sallusto and Lanzavecchia [28] using CD14^+^ peripheral blood mononuclear cells from healthy donors as detailed previously [87]. Volunteer donors gave written informed consent for the use of such material for research purposes as approved by the Ethics Committee of Fondazione IRCCS Policlinico San Matteo (Pavia, Italy). CD14^+^ cells were magnetically selected by means of CD14-labeled microbeads (Miltenyi Biotec, Bergisch Gladbach, Germany) according to the manufacturer's instructions. After 5 days incubation in differentiating medium (RPMI 1640 supplemented with 10% FBS and 2 mM glutamine and containing 500 U/ml human recombinant IL-4 (R&D Systems, Minneapolis, MN, USA) and 800 U/ml human recombinant GM-CSF (Schering–Plough/Sandoz, Basel, Switzerland), at least 80% of the cells were shown to express CD1a on their surface by flow cytometry (Navios flow cytometer equipped with Kaluza 1.1 software; Beckman Coulter, Brea, CA) using a specific FITC-labeled monoclonal antibody (BD Pharmingen, San Jose, CA).

Human NK cells were purified from peripheral blood mononuclear cells from healthy volunteer donors, using the NK Cell Isolation Kit (Miltenyi Biotec; based on depletion of magnetically labeled cells) according to the manufacturer's instructions. At least 90% of the cells were shown to be CD56^+^/CD3^−^ on their surface by flow cytometry. NK cells were incubated overnight with RPMI 1640 supplemented with 5% pooled human serum and 2 mM glutamine, with or without human recombinant IL-2 (100 U/ml; Aldesleukin, Chiron, Emeryville, CA) or human recombinant IL-15 (10 ng/ml; R&D Systems), as described by Pende et al. [27].

### Antibodies

The following primary antibodies were used: mouse monoclonal anti-LAMP1 (CD107a; BD Bioscience, Franklin Lakes, NJ), mouse monoclonal anti-chondroitin sulfate (CS-56; Sigma–Aldrich), mouse monoclonal anti-polyubiquitinated proteins (FK1 clone) and rabbit polyclonal anti-20S proteasome core subunits (Enzo Life Sciences International, Plymouth Meeting, PA), rabbit polyclonal anti-20S proteasome core subunits and rabbit polyclonal anti-19S proteasome S2-subunit (Calbiochem, Merck–Millipore, Darmstadt, Germany), rabbit polyclonal and mouse monoclonal anti-p62 (Santa Cruz Biotechnology, Santa Cruz, CA), mouse monoclonal anti-glycogen [21] kindly provided by Dr. O. Baba (Tokyo, Japan), rabbit monoclonal anti-glycogen synthase (Epitomics, Burlingame, CA), rabbit polyclonal anti-ubiquitin (Dako, Glostrup, Denmark), and rabbit polyclonal anti-ALFY (WDFY3; Novus Biologicals, Littleton, CO).

As secondary antibodies for confocal microscopy we used: Alexa-488-labeled anti-mouse IgG or anti-rabbit IgG (Life Technologies, Paisley, UK), aminomethylcoumarin-acetate-labeled anti-mouse IgG/IgM, DyLight-488-labeled anti-mouse IgM, Texas-Red-labeled anti-mouse IgG, and Cy5-labeled anti-rabbit IgG (all from Jackson Immunoresearch, West Grove, PA). For ultrastructural immunocytochemistry, we used: anti-rabbit or anti-mouse IgG or IgM secondary antibodies labeled with colloidal gold particles (5–20 nm diameter) (British BioCell, Cardiff, UK; and Aurion, Wageningen, Netherlands).

Primary antibodies used for immunogold procedures were selected among a larger antibody panel that was already found to work (and tested for specificity) under light microscopy on paraffin-embedded sections or in confocal microscopy preparations [15]–[17]. Specificity tests for the immunogold procedure included: (1) substitution of the specific primary antibody with pertinent non-immune Ig, at a 5–10-fold higher concentration, in the first layer of the procedure; (2) primary antibodies previously adsorbed with the pertinent purified antigen; and (3) negative and positive controls, represented by structures of known reactivity, in the same or different sections run in parallel.

### TEM and ultrastructural immunocytochemistry

For TEM, the cells were pelleted and fixed for 1–4 h at 4°C with 2.5% glutaraldehyde and 2% formaldehyde in 0.2 M cacodylate buffer (pH 7.3), followed by 1.5% osmium tetroxide for 1 h at room temperature, or they were fixed for 1 h at 4°C in a freshly prepared mixture of one part 2.5% glutaraldehyde and two parts 1% osmium tetroxide in cacodylate buffer, as described by Hirsch and Fedorko [88]. After dehydration in ethanol and propylene oxide, the specimens were embedded in Epon–Araldite resin. Semithin (∼1 µm) resin sections were stained with toluidine blue in a pH 8.0 borax solution [16], and thin (∼70 nm) sections were stained with uranyl–lead or underwent immunogold procedures followed by uranyl–lead staining [15]. Specimens were analyzed by a Jeol JEM-1200 EX II transmission electron microscope equipped with an Olympus CCD camera Mega View III. In several cases, a toluidine blue-stained semithin section was analyzed comparatively with a consecutive thin section, with or without immunogold tests, and viewed by TEM.

### Confocal microscopy immunocytochemistry

After washing, subconfluent cell monolayers or cell suspensions were: (1) fixed with 4% paraformaldehyde for 15 min at room temperature, washed three times in PBS, and treated with 50 mM NH_4_Cl in PBS for 5 min to quench free aldehyde groups (standard procedure); (2) fixed and processed as for TEM and embedded in Epon–Araldite resin or paraffin; and (3) fixed/permeabilized in methanol for 5 min at −20°C followed by acetone for 30 s at −20°C.

After washing with PBS (and for paraformaldehyde-fixed samples, permeabilization with PBS containing 0.5% BSA and 0.5% saponin for 5 min), samples were incubated for 1 h at room temperature, first with primary antibody and then with fluorescent secondary antibody as previously described [89]. When necessary, Hoechst 33258 was used for nuclear counterstaining. A TCS SP5II confocal laser scanning microscope equipped with PL APO 40×/1.25 NA and 63×/1.40 NA oil-immersion objectives (Leica, Heidelberg, Germany) was used. After acquisition, images were processed using Leica LAS Lite 3.1.8587.0 image analysis software and then Adobe Photoshop software (Adobe Systems, San Jose, CA).

### Correlative confocal/electron microscopy

For correlative confocal/electron microscopy, the two faces of a thin resin section collected on a 200-mesh Gilder Finder grid (Electron Microscopy Sciences, Hatfield, PA) were processed separately, as described previously [15], [16]. First, one face was immunostained and viewed by confocal microscopy as above after mounting the grid in 50% glycerol between a glass slide and a coverslip. Then the grid was removed from the mounting medium, extensively washed with PBS, and the reverse face of the section was processed for immunogold labeling and observed by TEM after uranyl–lead staining. The resulting confocal and TEM images of the same area were then overlapped using Adobe Photoshop software.

### Proteasome activity assay in living cells

Proteasome activity was assessed in living cells using the TAT-EDANS-DABCYL (TED) peptide. TED is a recently engineered, cell-penetrating, internally-quenched fluorogenic peptide with a proteasome-specific cleavage motif fused to TAT and linked to the fluorophores DABCYL and EDANS [25]. TED is specifically recognized and hydrolyzed in a ubiquitination-independent fashion by the 20S proteasome chymotrypsin-like activity, thus generating (through the physical separation of the DABCYL/EDANS pair that removes their intramolecular quenching, thus producing an increase in EDANS fluorescence proportional to the amount of substrate cleaved) a fluorescent reporter of proteasome activity *in vivo* [25].

After triple rinsing with HBSS, subconfluent HeLa cells grown in glass-bottomed dishes (Ibidi, Martinsried, Germany) were incubated at 37°C with HBSS containing 10 µM TED. After variable periods of time (5–50 min), the EDANS-dependent fluorescent signal was immediately acquired by confocal microscopy. To ascertain the specific role of proteasome in generating such a fluorescent signal, paired dishes of HeLa cells were treated for 6 h with the selective proteasome inhibitor epoxomicin (Sigma–Aldrich; 4 µM in cell growth medium), before being incubated with TED (in the continuous presence of epoxomicin) and then analyzed by confocal microscopy.

For correlative confocal/electron microscopy, after confocal microscopy acquisition of TED-generated images, living cells on gridded glass-bottomed dishes were immediately fixed at 4°C for 1 h with 2.5% glutaraldehyde and 2% formaldehyde in 0.2 M cacodylate buffer (pH 7.3), followed by 1.5% osmium tetroxide for 1 h at room temperature. The specimens were then embedded in Epon–Araldite resin, processed for ultrastructural immunocytochemistry, and analyzed by TEM. Confocal and TEM images of the same area were paired using Adobe Photoshop software.

## References

[1] Kuusisto E, Salminen A, Alafuzoff I (2001) Ubiquitin-binding protein p62 is present in neuronal and glial inclusions in human tauopathies and synucleinopathies. Neuroreport 12: 2085–2090. 11447312 10.1097/00001756-200107200-00009

[2] Willis MS, Patterson C (2013) Proteotoxicity and cardiac dysfunction – Alzheimer's disease of the heart? N Engl J Med 368: 455–464. 23363499 10.1056/NEJMra1106180

[3] Zatloukal K, Stumptner C, Fuchsbichler A, Heid H, Schnoelzer M, et al (2002) p62 is a common component of cytoplasmic inclusion in protein aggregation diseases. Am J Pathol 160: 255–263. 11786419 10.1016/S0002-9440(10)64369-6 PMC1867135

[4] Johnston JA, Ward CL, Kopito RR (1998) Aggresomes: a cellular response to misfolded proteins. J Cell Biol 143: 1883–1898. 9864362 10.1083/jcb.143.7.1883 PMC2175217

[5] Kopito RR (2000) Aggresomes, inclusion bodies and protein aggregation. Trends Cell Biol 10: 524–530. 11121744 10.1016/s0962-8924(00)01852-3

[6] Szeto J, Kaniuk NA, Canadien V, Nisman R, Mizushima N, et al (2006) ALIS are stress-induced protein storage compartments for substrates of the proteasome and autophagy. Autophagy 2: 189–199. 16874109 10.4161/auto.2731

[7] Lelouard H, Ferrand V, Marguet D, Bania J, Camossetto V, et al (2004) Dendritic cell aggresome-like induced structures are dedicated areas for ubiquitination and storage of newly synthesized defective proteins. J Cell Biol 164: 667–675. 14981091 10.1083/jcb.200312073 PMC2172164

[8] Lelouard H, Gatti E, Cappello F, Gresser O, Camossetto V, et al (2002) Transient aggregation of ubiquitinated proteins during dendritic cell maturation. Nature 417: 177–182. 12000969 10.1038/417177a

[9] Canadien V, Tan T, Zilber R, Szeto J, Perrin AJ, et al (2005) Cutting edge: microbial products elicit formation of dendritic cell aggresome-like induced structures in macrophages. J Immunol 174: 2471–2475. 15728449 10.4049/jimmunol.174.5.2471

[10] Bjørkøy G, Lamark T, Brech A, Outzen H, Perander M, et al (2005) P62/SQSTM1 forms protein aggregates degraded by autophagy and has a protective effect on huntingtin-induced cell death. J Cell Biol 171: 603–614. 16286508 10.1083/jcb.200507002 PMC2171557

[11] Pankiv S, Clausen TH, Lamark T, Brech A, Bruun JA, et al (2007) p62/SQSTM1 binds directly to Atg8/LC3 to facilitate degradation of ubiquitinated protein aggregates by autophagy. J Biol Chem 282: 24131–24145. 17580304 10.1074/jbc.M702824200

[12] Clausen TH, Lamark T, Isakson P, Finley K, Larsen KB, et al (2010) P62/SQSTM1 and ALFY interact to facilitate the formation of p62 bodies/ALIS and their degradation by autophagy. Autophagy 6: 330–344. 20168092 10.4161/auto.6.3.11226

[13] Kirkin V, Lamark T, Sou Y-S, Bjørkøy G, Nunn JL, et al (2009) A role for NBR1 in autophagosomal degradation of ubiquitinated substrates. Mol Cell 33: 505–516. 19250911 10.1016/j.molcel.2009.01.020

[14] Kaganovich D, Kopito R, Frydman J (2008) Misfolded proteins partition between two distinct quality control compartments. Nature 454: 1088–1095. 18756251 10.1038/nature07195 PMC2746971

[15] Necchi V, Sommi P, Ricci V, Solcia E (2010) *In vivo* accumulation of *Helicobacter pylori* products, NOD1, ubiquitinated proteins and proteasome in a novel cytoplasmic structure. PLoS ONE 5: e9716. 20300534 10.1371/journal.pone.0009716 PMC2838800

[16] Necchi V, Sommi P, Vanoli A, Manca R, Ricci V, et al (2011) Proteasome particle-rich structures are widely present in human epithelial neoplasms: correlative light, confocal and electron microscopy study. PLoS ONE 6: e21317. 21695063 10.1371/journal.pone.0021317 PMC3117888

[17] Necchi V, Minelli A, Sommi P, Vitali A, Caruso R, et al (2012) Ubiquitin-proteasome-rich cytoplasmic structures in neutrophils of patients with Shwachman-Diamond syndrome. Haematologica 97: 1057–1063. 22271888 10.3324/haematol.2011.048462 PMC3396678

[18] Necchi V, Balduini A, Noris P, Barozzi S, Sommi P, et al (2013) Ubiquitin/proteasome-rich particulate cytoplasmic structures (PaCSs) in the platelets and megakaryocytes of ANKRD26-related thrombocytopenia. Thromb Haemost 109: 263–271. 23223974 10.1160/TH12-07-0497

[19] Gray CW, Slaughter CA, DeMartino GN (1994) PA28 activator protein forms regulatory caps on proteasome stacked rings. J Mol Biol 236: 7–15. 8107126 10.1006/jmbi.1994.1113

[20] Fujimuro M, Sawada H, Yokosawa H (1994) Production and characterization of monoclonal antibodies specific to multi-ubiquitin chains of polyubiquitinated proteins. FEBS Lett 349: 173–180. 7519568 10.1016/0014-5793(94)00647-4

[21] Baba O (1993) Production of monoclonal antibody that recognizes glycogen and its application for immunohistochemistry. Kokubyo Gakkai Zasshi 60: 264–287. 8345245 10.5357/koubyou.60.264

[22] Hudson ER, David AP, James J, Lucocq JM, Hawley SA, et al (2003) A novel domain in AMP-activated protein kinase causes glycogen storage bodies similar to those seen in hereditary cardiac arrhythmias. Curr Biol 13: 861–866. 12747836 10.1016/s0960-9822(03)00249-5

[23] Simonsen A, Birkeland HCG, Gillooly DJ, Mizushima N, Kuma A, et al (2004) Alfy, a novel FYVE-domain-containing protein associated with protein granules and autophagic membranes. J Cell Sci 117: 4239–4251. 15292400 10.1242/jcs.01287

[24] Shearer J, Graham TE (2002) New perspectives on the storage and organization of muscle glycogen. Can J Appl Physiol 27: 179–203. 12179957 10.1139/h02-012

[25] Urru SAM, Veglianese P, De Luigi A, Fumagalli E, Erba E, et al (2010) A new fluorogenic peptide determines proteasome activity in single cells. J Med Chem 53: 7452–7460. 20883027 10.1021/jm100362x

[26] Nannmark U, Kitson RP, Johansson BR, Rivett J (1996) Immunocytochemical localization of multicatalytic protease complex (proteasome) during generation of murine IL-2-activated natural killer (A-NK) cells. Eur J Cell Biol 71: 402–408. 8980912

[27] Pende D, Marcenaro S, Falco M, Martini S, Bernardo ME, et al (2009) Anti-leukemia activity of alloreactive NK cells in KIR ligand-mismatched haploidentical HSCT for pediatric patients: evaluation of the functional role of activating KIR and redefinition of inhibitory KIR specificity. Blood 113: 3119–3129. 18945967 10.1182/blood-2008-06-164103

[28] Sallusto F, Lanzavecchia A (1994) Efficient presentation of soluble antigen by cultured human dendritic is maintained by granulocyte/macrophage colony stimulating factor plus interleukin 4 and downregulated by tumor necrosis factor alpha. J Exp Med 179: 1109–1118. 8145033 10.1084/jem.179.4.1109 PMC2191432

[29] Denk H, Stumptner C, Fuchsbichler A, Müller T, Farr GH, et al (2006) Are the Mallory bodies and intracellular hyaline bodies in neoplastic and non-neoplastic hepatocytes related? J Pathol 208: 653–661. 16477590 10.1002/path.1946

[30] Harper JD, Wong SS, Lieber CM, Lansbury PT Jr (1999) Assembly of Aβ amyloid protofibrils: an in vitro model for a possible early event in Alzheimer's disease. Biochemistry 38: 8972–8980. 10413470 10.1021/bi9904149

[31] Walsh DM, Hartley DM, Kusumoto Y, Fezoui Y, Condron MM, et al (1999) Amyloid β-protein fibrillogenesis. Structure and biological activity of protofibrillar intermediates. J Biol Chem 274: 25945–25952. 10464339 10.1074/jbc.274.36.25945

[32] Goldberg MS, Lansbury PT Jr (2000) Is there a cause-and-effect relationship between α-synuclein fibrillization and Parkinson's disease? Nat Cell Biol 2: E115–E119. 10878819 10.1038/35017124

[33] Scherzinger E, Sittler A, Schweiger K, Heiser V, Lurz R, et al (1999) Self-assembly of polyglutamine-containing huntingtin fragments into amyloid-like fibrils: implications for Huntington's disease pathology. Proc Natl Acad Sci USA 96: 4604–4609. 10200309 10.1073/pnas.96.8.4604 PMC16379

[34] Hartley DM, Walsh DM, Ye CP, Diehl T, Vasquez S, et al (1999) Protofibrillar intermediates of amyloid β-protein induce acute electrophysiological changes and progressive neurotoxicity in cortical neurons. J Neurosci 19: 8876–8884. 10516307 10.1523/JNEUROSCI.19-20-08876.1999 PMC6782787

[35] Kayed R, Head E, Sarsoza F, Saing T, Cotman CW, et al (2007) Fibril specific, conformation dependent antibodies recognize a generic epitope common to amyloid fibrils and fibrillar oligomers that is absent in prefibrillar oligomers. Mol Neurodegener 2: 18. 17897471 10.1186/1750-1326-2-18 PMC2100048

[36] Kayed R, Head E, Thompson JL, McIntire TM, Milton SC, et al (2003) Common structure of soluble amyloid oligomers implies common mechanism of pathogenesis. Science 300: 486–489. 12702875 10.1126/science.1079469

[37] Alonso LG, García-Alai MM, Smal C, Centeno JM, Iacono R, et al (2004) The HPV16 E7 viral oncoprotein self-assembles into defined spherical oligomers. Biochemistry 43: 3310–3317. 15035602 10.1021/bi036037o

[38] Dantur K, Alonso L, Castaño E, Morelli L, Centeno-Crowley JM, et al (2009) Cytosolic accumulation of HPV16 E7 oligomers supports different transformation routes for the prototypic viral oncoprotein: the amyloid-cancer connection. Int J Cancer 125: 1902–1911. 19598264 10.1002/ijc.24579

[39] Nishimura A, Nakahara T, Ueno T, Sasaki K, Yoshiba S, et al (2006) Requirement of E7 oncoprotein for viability of HeLa cells. Microbes Infect 8: 984–993. 16500131 10.1016/j.micinf.2005.10.015

[40] Lamark T, Perander M, Outzen H, Kristiansen K, Øvervatn A, et al (2003) Interaction codes within the family of mammalian Phox and Bem1p domain-containing proteins. J Biol Chem 278: 34568–34581. 12813044 10.1074/jbc.M303221200

[41] Riley BE, Kaiser SE, Shaler TA, Ng AC, Hara T, et al (2010) Ubiquitin accumulation in autophagy-deficient mice is dependent on the Nrf2-mediated stress response pathway: a potential role for protein aggregation in autophagic substrate selection. J Cell Biol 191: 537–552. 21041446 10.1083/jcb.201005012 PMC3003313

[42] Itakura E, Mizushima N (2011) p62 targeting to the autophagosome formation site requires self-oligomerization but not LC3 binding. J Cell Biol 192: 17–27. 21220506 10.1083/jcb.201009067 PMC3019556

[43] Hara T, Nakamura K, Matsui M, Yamamoto A, Nakahara Y, et al (2006) Suppression of basal autophagy in neural cells causes neurodegenerative disease in mice. Nature 441: 885–889. 16625204 10.1038/nature04724

[44] Wenger T, Terawaki S, Camosseto V, Abdelrassoul R, Mies A, et al (2012) Autophagy inhibition promotes defective neosynthesized proteins storage in ALIS, and induces redirection toward proteasome processing and MHCI-restricted presentation. Autophagy 8: 350–363. 22377621 10.4161/auto.18806

[45] Pearse AGE (1985) Histochemistry, Theoretical and Applied. In Vol. 2: Analytical Technology. Churchill Livingstone, Edinburgh. 1055 pp.

[46] Kuboki Y, Shiratori K, Hatori T, Fujita I, Kimijima A, et al (2010) Association of epidermal growth factor receptor and mitogen-activated protein kinase with cystic neoplasm of the pancreas. Mod Pathol 23: 1127–1135. 20495538 10.1038/modpathol.2010.97

[47] Keates S, Keates AC, Katchar K, Peek RM Jr, Ciarán PK (2007) *Helicobacter pylori* induces up-regulation of the epidermal growth factor receptor in AGS gastric epithelial cells. J Infect Dis 196: 95–103. 17538889 10.1086/518440

[48] Meiners S, Heyken D, Weller A, Ludwig A, Stangl K, et al (2003) Inhibition of proteasome activity induces concerted expression of proteasome genes and *de novo* formation of mammalian proteasomes. J Biol Chem 278: 21517–21525. 12676932 10.1074/jbc.M301032200

[49] Bazzaro M, Lee MK, Zoso A, Stirling WLH, Santillan A, et al (2006) Ubiquitin-proteasome system stress sensitizes ovarian cancer to proteasome inhibitor-induced apoptosis. Cancer Res 66: 3754–3763. 16585202 10.1158/0008-5472.CAN-05-2321

[50] Bennett EJ, Bence NF, Jayakumar R, Kopito RR (2005) Global impairment of the ubiquitin-proteasome system by nuclear or cytoplasmic protein aggregates precedes inclusion body formation. Mol Cell 17: 351–365. 15694337 10.1016/j.molcel.2004.12.021

[51] Chen Q, Liu J-B, Horak KM, Zheng H, Kumarapeli ARK, et al (2005) Intrasarcoplasmic amyloidosis impairs proteolytic fuction of proteasomes in cardiomyocytes by compromising substrate uptake. Circ Res 97: 1018–1026. 16210548 10.1161/01.RES.0000189262.92896.0b

[52] Baumeister W, Dahlmann B, Hegerl R, Kopp F, Kuehn L, et al (1988) Electron microscopy and image analysis of the multicatalytic proteinase. FEBS Lett 241: 239–245. 2461878 10.1016/0014-5793(88)81069-x

[53] Walz J, Erdmann A, Kania M, Typke D, Koster AJ, et al (1998) 26S proteasome structure revealed by three-dimensional electron microscopy. J Struct Biol 121: 19–29. 9573617 10.1006/jsbi.1998.3958

[54] Wasserman K, Kitson RP, Rivett AJ, Sweeney ST, Gabauer MK, et al (1994) Nongranular proteolytic enzymes of rat IL-2-activated natural killer cells. II. Purification and identification of rat A-NKP 1 and A-NKP 2 as constituents of the multicatalytic proteinase (proteasome) complex. J Cell Biochem 55: 133–145. 8083294 10.1002/jcb.240550115

[55] Finley D (2009) Recognition and processing of ubiquitin-protein conjugates by the proteasome. Annu Rev Biochem 78: 477–513. 19489727 10.1146/annurev.biochem.78.081507.101607 PMC3431160

[56] Peth A, Besche HC, Goldberg AL (2009) Ubiquitinated proteins activate the proteasome by binding to USP14/UBP6 which causes 20S gate opening. Mol Cell 36: 794–804. 20005843 10.1016/j.molcel.2009.11.015 PMC2796264

[57] Baugh JM, Viktorova EG, Pilipenko EV (2009) Proteasomes can degrade a significant proportion of cellular proteins independent of ubiquitination. J Mol Biol 386: 814–827. 19162040 10.1016/j.jmb.2008.12.081 PMC2649715

[58] Liu C-W, Corboy MJ, DeMartino GN, Thomas PJ (2003) Endoproteolytic activity of the proteasome. Science 299: 408–411. 12481023 10.1126/science.1079293 PMC3516294

[59] Pickering AM, Koop AL, Teoh CY, Ermark G, Grune T, et al (2010) The immunoproteasome, the 20S proteasome, and the PA28αβ proteasome regulator are oxidative-stress-adaptive proteolytic complexes. Biochem J 432: 585–594. 20919990 10.1042/BJ20100878 PMC3133595

[60] Fujita K-i, Maeda D, Xiao Q, Srinivasula SM (2011) Nrf2-mediated induction of p62 controls Toll-like receptor-4–driven aggresome-like induced structure formation and autophagic degradation. Proc Natl Acad Sci USA 108: 1427–1432. 21220332 10.1073/pnas.1014156108 PMC3029726

[61] Qureshi N, Perera P-Y, Shen J, Zhang G, Lenschat A, et al (2003) The proteasome as a lipopolysaccharide-binding protein in macrophages: differential effects of proteasome inhibition on lipopolysaccharide-induced signaling events. J Immunol 171: 1515–1525. 12874245 10.4049/jimmunol.171.3.1515

[62] Dhungana S, Merrick BA, Tomer KB, Fessler MB (2009) Quantitative proteomics analysis of macrophage rafts reveals compartmentalized activation of the proteasome and of proteasome-mediated ERK activation in response to lipopolysaccharide. Mol Cell Proteomics 8: 201–213. 18815123 10.1074/mcp.M800286-MCP200 PMC2621002

[63] Rivett AJ (1998) Intracellular distribution of proteasomes. Curr Opin Immunol 10: 110–114. 9523120 10.1016/s0952-7915(98)80040-x

[64] Segundo C, Medina F, Rodríguez C, Martínez-Palencia R, Levyva-Cobiá F, et al (1999) Surface molecule loss and bleb formation by human germinal center B cells undergoing apoptosis: role of apoptotic blebs in monocyte chemotaxis. Blood 94: 1012–1020. 10419893

[65] Suber T, Rosen A (2009) Apoptotic cell blebs: repositories of autoantigens and contributors to immune context. Arthritis Rheum 60: 2216–2219. 19644864 10.1002/art.24715 PMC3638860

[66] Pitzer F, Dantes A, Fuchs T, Baumeister W, Amsterdam A (1996) Removal of proteasomes from the nucleus and their accumulation in apoptotic bleds during programmed cell death. FEBS Lett 394: 47–50. 8925925 10.1016/0014-5793(96)00920-9

[67] Ohkawa K, Amasaki H, Terashima Y, Aizawa S, Ishikawa E (1977) Clear cell carcinoma of the ovary. Cancer 40: 3019–3029. 589565 10.1002/1097-0142(197712)40:6<3019::aid-cncr2820400639>3.0.co;2-m

[68] Mittal S, Dubey D, Yamakawa K, Ganesh S (2007) Lafora disease proteins malin and laforin are recruited to aggresomes in response to proteasomal impairment. Hum Mol Genet 16: 753–762. 17337485 10.1093/hmg/ddm006

[69] Worby CA, Gentry MS, Dixon JE (2008) Malin decreases glycogen accumulation by promoting the degradation of protein targeting to glycogen (PTG). J Biol Chem 283: 4069–4076. 18070875 10.1074/jbc.M708712200 PMC2251628

[70] Solaz-Fuster MC, Gimeno-Alcañiz JV, Ros S, Fernandez-Sanchez ME, Garcia-Fojeda B, et al (2008) Regulation of glycogen synthesis by the laforin-malin complex is modulated by the AMP-activated protein kinase pathway. Hum Mol Genet 17: 667–678. 18029386 10.1093/hmg/ddm339

[71] Garyali P, Siwach P, Singh PK, Puri R, Mittal S, et al (2009) The malin-laforin complex suppresses the cellular toxicity of misfolded proteins by promoting their degradation through the ubiquitin-proteasome system. Hum Mol Genet 18: 688–700. 19036738 10.1093/hmg/ddn398

[72] Puri R, Jain N, Ganesh S (2011) Increased glucose concentration results in reduced proteasomal activity and the formation of glycogen positive aggresomal structures. FEBS J 278: 3688–3698. 21815999 10.1111/j.1742-4658.2011.08287.x

[73] Cheng KW, Agarwal R, Mitra S, Lee JS, Carey M, et al (2012) Rab25 increases cellular ATP and glycogen stores protecting cancer cells from bioenergetic stress. EMBO Mol Med 4: 125–141. 22253197 10.1002/emmm.201100193 PMC3306554

[74] Lunt SY, Vander Heiden MG (2011) Aerobic glycolysis: meeting the metabolic requirements of cell proliferation. Annu Rev Cell Dev Biol 27: 441–464. 21985671 10.1146/annurev-cellbio-092910-154237

[75] Mani K, Cheng F, Frasson LÅ (2007) Heparan sulfate degradation products can associate with oxidized protein and proteasomes. J Biol Chem 282: 21934–21944. 17540770 10.1074/jbc.M701200200

[76] Ohkubo I, Gasa S, Namikawa C, Makita A, Sasaki M (1991) Human erythrocyte multicatalytic proteinase: activation and binding to sulfated galacto- and lactosylceramides. Biochem Biophys Res Commun 174: 1133–1140. 1825464 10.1016/0006-291x(91)91538-n

[77] Bao X, Nishimura S, Mikami T, Yamada S, Itoh N, et al (2004) Chondroitin sulfate/dermatan sulfate hybrid chains from embryonic pig brain, which contain a higher proportion of L-iduronic acid than those from adult pig brain, exhibit neuritogenic and growth factor binding activities. J Biol Chem 279: 9765–9776. 14699094 10.1074/jbc.M310877200

[78] Hatakeyama M (2004) Oncogenic mechanisms of the *Helicobacter pylori* CagA protein. Nature 4: 688–694. 10.1038/nrc1433 15343275

[79] Boocock GRB, Morrison JA, Popovic M, Richards N, Ellis L, et al (2003) Mutations in *SBDS* are associated with Shwachman-Diamond syndrome. Nat Genet 33: 97–101. 12496757 10.1038/ng1062

[80] Noris P, Perrotta S, Seri M, Pecci A, Gnan C, et al (2011) Mutations in *ANKRD26* are responsible for a frequent form of inherited thrombocytopenia: analysis of 78 patients from 21 families. Blood 117: 6673–6680. 21467542 10.1182/blood-2011-02-336537

[81] Pippucci T, Savoia A, Perrotta S, Pujol-Moix N, Noris P, et al (2011) Mutation in 5′ UTR of *ANKRD26*, in the ankirin repeat domain 26 gene, cause an autosomal-dominant form of inherited thrombocytopenia, THC2. Am J Hum Genet 88: 115–120. 21211618 10.1016/j.ajhg.2010.12.006 PMC3014357

[82] Hipp MS, Patel CN, Bersuker K, Riley BE, Kaiser SE, et al (2012) Indirect inhibition of 26S proteasome activity in a cellular model of Huntington's disease. J Cell Biol 196: 573–587. 22371559 10.1083/jcb.201110093 PMC3307690

[83] Fassbender M, Herter S, Holtapples R, Schild H (2008) Correlation of dendritic cell maturation and the formation of aggregates of poly-ubiquitinated proteins in the cytosol. Med Microbiol Immunol 197: 185–189. 18340462 10.1007/s00430-008-0091-4

[84] Herter S, Osterloh P, Hilf N, Rechtsteiner G, Höhfeld J, et al (2005) Dendritic cell aggresome-like-induced structure formation and delayed antigen presentation coincide in influenza virus-infected dendritic cells. J Immunol 175: 891–898. 16002687 10.4049/jimmunol.175.2.891

[85] Pierre P (2005) Dendritic cells, DRiPs, and DALIS in control af antigen processing. Immunol Rev 207: 184–190. 16181336 10.1111/j.0105-2896.2005.00300.x

[86] Chiozzi V, Mazzini G, Oldani A, Sciullo A, Ventura U, et al (2009) Relationship between VacA toxin and ammonia in *Helicobacter pylori*-induced apoptosis in human gastric epithelial cells. J Physiol Pharmacol 60: 23–30. 19826178

[87] Montagna D, Maccario R, Montini E, Tonelli R, Lisini D, et al (2003) Generation and ex vivo expansion of cytotoxic T lymphocytes directed towards different types of leukemia or myelodysplastic cells using both HLA-matched and partially matched donors. Exp Hematol 31: 1031–1038. 14585366 10.1016/s0301-472x(03)00230-3

[88] Hirsch JG, Fedorko ME (1968) Ultrastructure of human leukocytes after simultaneous fixation with glutaraldehyde and osmium tetroxide and “postfixation” in uranyl acetate. J Cell Biol 38: 615–627. 4874495 10.1083/jcb.38.3.615 PMC2108377

[89] Ricci V, Galmiche A, Doye A, Necchi V, Solcia E, et al (2000) High cell sensitivity to *Helicobacter pylori* VacA toxin depends on a GPI-anchored protein and is not blocked by inhibition of the clathrin-mediated pathway of endocytosis. Mol Biol Cell 11: 3897–3909. 11071915 10.1091/mbc.11.11.3897 PMC15045

